# Notch signaling pathway modulation by food functional ingredients: mechanisms, cancer and immunoregulation

**DOI:** 10.3389/fcell.2025.1729265

**Published:** 2026-01-12

**Authors:** Rong-Zu Nie, Huo-Min Luo, Ya-Ping Liu, Shuang-Shuang Wang, Yan-Jie Hou, Chen Chen, Hang Wang, Hui-Lin Lv, Xing-Yue Tao, Zhao-Hui Jing, Hao-Kun Zhang, Pei-Feng Li

**Affiliations:** 1 College of Food and Bioengineering, Zhengzhou University of Light Industry, Zhengzhou, China; 2 Henan Key Laboratory of Cold Chain Food Quality and Safety Control, Zhengzhou University of Light Industry, Zhengzhou, China; 3 Key Laboratory of Cold Chain Food Processing and Safety Control, Ministry of Education, Zhengzhou University of Light Industry, Zhengzhou, China

**Keywords:** anti-cancer mechanism, cancer treatment, food functional ingredients, immunomodulation, notch signaling

## Abstract

This article comprehensively reviews the complex role of the Notch signaling cascade in cancer, as well as the regulatory mechanisms and potential anti-cancer effects of food functional ingredients on this pathway. The Notch signaling cascade is essential for maintaining normal cellular physiological processes and is closely associated with cancer initiation and progression; its abnormalities are linked to diverse biological behaviors of tumor cells, and it exhibits dual roles in both pro-cancer and anti-cancer properties. Food functional ingredients, including polyphenols, terpenoids, sulfur-containing compounds, vitamins, minerals, and other compounds, can precisely regulate the Notch signaling pathway *via* distinct molecular mechanisms and exert significant anti-cancer activity. *In vitro* cell experiments have elucidated the regulatory effects of these ingredients on the Notch pathway and their impacts on cancer cell phenotypes at the molecular level, while *in vivo* animal experiments further verified their efficacy in inhibiting tumor growth and metastasis. Although clinical research remains in its infancy, existing studies have provided directions for subsequent basic and clinical investigations-specifically, to further clarify the detailed mechanisms by which functional ingredients in food regulate the Notch signaling cascade and facilitate their clinical application in cancer treatment.

## 1 Introduction

Cancer is a complex disease caused by genomic changes and poses a serious threat to human health and life. Its pathogenesis involves abnormal changes at multiple levels, including gene mutations, epigenetic modifications, and signaling pathway disorders (Park et al., 2011). Among them, the Notch signaling pathway-a highly conserved signaling mechanism throughout evolution-plays a critical role in processes such as cell differentiation, proliferation, apoptosis, and stem cell maintenance, thereby garnering significant attention in cancer research (Shi et al., 2024; Remark et al., 2023). Notably, this pathway exhibits a context-dependent dual role in cancer. It can promote tumor cell proliferation, invasion, and stemness maintenance, or it can suppress tumor progression by modulating the tumor microenvironment. However, the subtype-specific regulatory principles underlying this dual function, as well as the downstream target gene networks in different cancer types, have not been systematically integrated, resulting in ambiguity in the development of targeted intervention strategies.

At the same time, food functional ingredients, as a class of chemical substances with specific biological activities that exist naturally in food, are gradually becoming a new focus in the field of cancer research. These ingredients include polyphenols, terpenoids, sulfur-containing compounds, vitamins and minerals, which have demonstrated promising potential in cancer prevention and therapy due to their diverse biological properties, such as antioxidant, anti-inflammatory, anti-proliferative, and immunomodulatory activities. Despite growing evidence that food functional ingredients can modulate the Notch signaling pathway to exert anti-cancer effects, several important knowledge gaps remain. First, most existing studies examine a single ingredient or rely on a specific cancer model, resulting in a lack of cross-category synthesis regarding the regulatory patterns of diverse food functional ingredients on the Notch pathway. Second, the crosstalk between Notch modulation by these ingredients and other cancer-related signaling axes (e.g., PI3K/Akt, NF-κB) is still insufficiently characterized. Third, the translational potential of these ingredients, including optimal dosage, routes of administration, and combination strategies with conventional anti-cancer therapies, has not been systematically evaluated, which continues to hinder the integration of basic findings into clinical practice. With the rapid development of modern research technologies, an increasing number of studies are devoted to revealing the intrinsic connection between food functional ingredients and cancer, especially their regulatory effects on the Notch signaling pathway, which has introduced a new, safe, and sustainable approach to cancer treatment based on dietary intervention.

This review will systematically elucidate the composition and signaling mechanisms of the Notch pathway, comprehensively examine its involvement in various cancer types, analyze in detail the molecular mechanisms through which food functional ingredients modulate this pathway, and summarize the *in vitro* and *in vivo* research results regarding how food functional ingredients regulate the Notch signaling pathway to exert anti-cancer effects. To ensure rigor, studies included in this review were selected based on their mechanistic relevance to Notch signaling, strength of experimental evidence, and direct association with cancer-related models. Based on this foundation, this review focuses on the role of food functional components in cancer prevention and treatment through the regulation of the Notch signaling pathway. Furthermore, this review identifies non-small cell lung cancer, breast cancer, colorectal cancer, and T-ALL (a hematological malignancy) as the cancer types with the most promising translational potential for Notch-targeted intervention, thereby providing direction for targeted dietary strategies and clinical applications. Overall, it aims to provide a comprehensive and in-depth theoretical basis for cancer treatment research and promote further development in this field.

## 2 Overview of the Notch signaling pathway and food functional ingredients

### 2.1 Notch signaling pathway

The Notch signaling pathway is an evolutionarily conserved cellular signaling system that plays a crucial role in regulating cell differentiation, proliferation, apoptosis, and stem cell maintenance (Shi et al., 2024; Remark et al., 2023). In the context of cancer, its function is notably complex. On the one hand, it can facilitate tumor cell proliferation and invasion; on the other hand, it may suppress tumor development by modulating the tumor microenvironment (Anusewicz et al., 2021). This dual functionality not only presents opportunities for targeting the Notch pathway in cancer therapy but also brings challenges.

**FIGURE 1:**
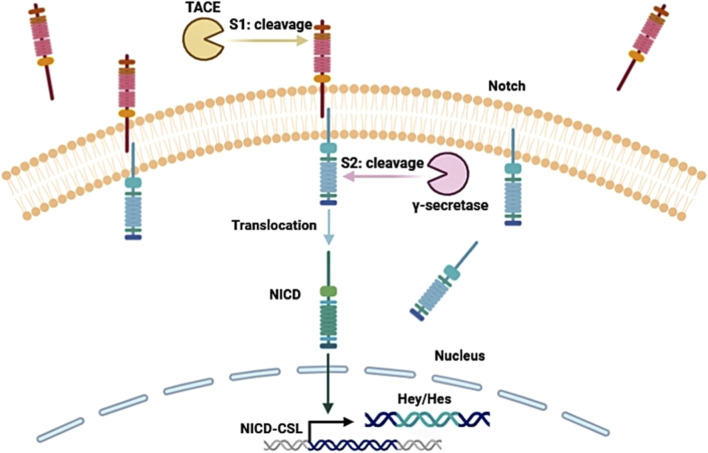
Schematic diagram of the Notch signaling pathway transduction mechanism. This figure illustrates the canonical activation process of the Notch signaling pathway, including ligand binding, sequential cleavage by TACE and γ-secretase, release and nuclear translocation of NICD, and the subsequent activation of CSL-dependent transcription of Hey/Hes target genes relevant to cancer regulation.

In recent years, increasing attention has been paid to the Notch signaling pathway due to its critical function in regulating cell fate. This pathway is a highly conserved intercellular communication system composed of Notch1-4 receptor family, Delta-like (DLL1/3/4) and Jagged (JAG1/2) ligands, CSL transcription factors (CBF1/RBP-Jκ, Su(H), LAG-1), and the regulatory proteases tumor necrosis factor-α converting enzyme (TACE) and γ-secretase (Table 1) (Zhai et al., 2023). Ligand-receptor binding triggers a highly dynamic cascade involving sequential cleavage by TACE and γ-secretase, leading to the release of the Notch intracellular domain (NICD) (Figure 1) (Zhuang et al., 2022). The nuclear transport of NICD is precisely regulated by phosphorylation, and once in the nucleus, NICD forms a complex with CSL to activate canonical Hes/Hey target genes and modulate chromatin-level gene regulation through interactions with epigenetic factors (Shi et al., 2024; Zhou et al., 2022; Braunreiter and Cole, 2019). Under normal physiological conditions, the Notch pathway maintains tissue homeostasis by tightly controlling cell differentiation and proliferation, whereas in cancer its aberrant activation or suppression contributes to tumor progression, metastasis, stemness, recurrence, and therapeutic resistance in a context-dependent manner (Shi et al., 2024).

**TABLE 1:** Functional mechanisms of signaling components in the Notch pathway.

Key ingredients	Signaling components	Main functional mechanism	References
Notch receptors	Notch1-4	Notch1: maintains tumor stemness and promote proliferation; Notch2: regulates cell differentiation and survival; Notch3: maintains vascular smooth muscle cell stability and promote angiogenesis; Notch4: regulates endothelial cell function and the response to tumor treatment	Huang et al. (2016), Manokawinchoke et al. (2020), Liu et al. (2010), Xiu et al. (2021)
Notch ligands	Delta-like 1, 3, 4, Jagged 1, 2	DLL1: regulates cell differentiation and tissue homeostasis; DLL3: regulates neural differentiation and developmental processes; DLL4: regulates angiogenesis and endothelial cell differentiation; JAG1: regulate cell-cell communication and stem cell fate; JAG2: regulates immune regulation and tissue development	Negri et al. (2019), Suchting et al. (2007), Karanu et al. (2000), Putti et al. (2019), Mandula et al. (2024)
Transcription factors	CBF1/RBP- Jκ, Su(H), LAG-1	CBF1/RBP-Jκ: acts as the core transcription factor of Notch signal transductions; Su(H): regulates Notch target gene expression; LAG-1: functions as the core element of Notch pathway transcriptional regulation	Maicas et al. (2021), Xu et al. (2017), Wolf et al. (2019)
Regulatory molecules	TACE, γ-secretase	TACE: cleaves and activates the extracellular domain (ECD) of the Notch receptors; γ-secretase: releases the intracellular domain (ICD) of the Notch receptors and mediates signal transduction	Delwig and Rand (2008), Guo et al. (2024a)

### 2.2 Food functional ingredients

With the continuous advancement of scientific research, food functional ingredients have gradually become a highly regarded research direction in cancer studies. They not only provide rich natural resources for the development of new cancer prevention strategies and auxiliary treatment approaches, but also provide new strategies for targeted cancer treatment. These food functional ingredients are expected to play a unique and important role in cancer treatment through dietary intervention, which is a safe and sustainable way (Table 2) (Abuajah et al., 2015). Therefore, the study of food functional ingredients is not only crucial for understanding their potential impact on cancer, but also to bring new hope for mankind to conquer this stubborn disease.

**TABLE 2:** Anticancer effects of food functional ingredients and regulation of Notch signaling pathway.

Food functional ingredients	Natural source	Regulatory mechanism related to Notch pathway	Role in cancer treatment	References
Epigallocatechin gallate	Tea	Inhibit Notch target gene expression and inflammatory response-related enzymes, and regulate the Jagged1/Notch pathway	Promote wound healing, reduce inflammation, suppress colorectal cancer and nasopharyngeal carcinoma tumors, and prevent infections	Xu et al. (2012), Li (2022), Wu et al. (2022)
Resveratrol	*Polygonum cuspidatum* rhizome	Inhibit Notch key molecules and block Notch-related axes, induce mitochondrial apoptosis	Suppress breast cancer and colorectal cancer cell proliferation and migration, and induce apoptosis in these cancer cells	Pandey et al. (2024), Kaveh Zenjanab et al. (2024), Ren et al. (2023), Chen et al. (2024)
Curcumin	Turmeric rhizome	Directly bind or indirectly inhibit protein kinases to affect Notch signaling, and regulate multiple pathways	Suppress T-ALL, colorectal cancer and cervical cancer tumor growth and metastasis, enhance chemotherapy efficacy for these cancers, and regulate nerve function	Liu et al. (2015), Zhdanovskaya et al. (2022), Ji et al. (2022), Li et al. (2019)
Quercetin	Fruits, vegetables and tea	Inhibit γ-secretase and regulate multiple pathways to affect Notch	Suppress Notch activity in breast, lung and colorectal cancer cells, induce apoptosis, regulate the tumor microenvironment, and enhance radiosensitivity of colorectal cancer	Cao et al. (2018), Gan et al. (2024), Das et al. (2023), Cai et al. (2024)
Ursolic acid	Apple, hawthorn and other fruit peels	Inhibit Notch pathway activation and related molecule expression and nuclear translocation	Suppress prostate cancer and gastric cancer cell proliferation and invasion, reduce fibrosis, and act through multiple anti-tumor mechanisms in these cancers	Zhang (2023), Chan et al. (2019)
Triptolide	*Tripterygium wilfordii*	Interfere with Notch’s interaction with other pathways to regulate cancer cell status	Reduce the proportion of T-ALL (leukemia) stem cells and induce cell differentiation	Wu et al. (2023)
Allicin	Garlic	Modulate redox, affect γ-secretase activity, and regulate Notch	Induce gastric, colorectal and breast cancer cell apoptosis, suppress tumor growth, and regulate immune and vascular function related to cancer progression	Zhang et al. (2019), Jin et al. (2023), Kiesel and Stan (2017)
Sulforaphane	Broccoli	Induce negative regulatory factors to inhibit Notch signaling	Suppress colorectal cancer cell growth and invasion, and improve the responsiveness to radiotherapy and chemotherapy	Wang M. et al. (2024)
Retinoic acid	Foods and supplements rich in vitamin A	Bind to nuclear receptors to indirectly regulate Notch gene expression	Induce salivary adenoid cystic carcinoma cell differentiation and reduce lung metastasis	Zhou et al. (2023)
Vitamin D	Vitamin D-rich foods and supplements	Regulate Notch receptor ligands and related factors to affect pathways	Suppress non-small cell lung cancer cell proliferation, migration, and invasion, and enhance synergy with chemotherapy	Domingues-Faria et al. (2014)
Selenium	Selenium-containing foods and mineral supplements	Regulate cellular redox to affect the activity and stability of Notch protein	Suppress breast and liver cancer cell proliferation, promote their apoptosis, enhance immunity, and indirectly inhibit tumor growth	Knight et al. (2016)
Zinc	Zinc-containing foods and zinc supplements	Stabilize or interfere with the structure and function of Notch receptors to affect transmission	Regulate non-small cell lung cancer and colorectal cancer cell proliferation, apoptosis, and metastasis	Scicchitano et al. (2023), Wang et al. (2023), Zhang et al. (2017)
Genistein	Soybeans and other legumes	Inhibit Notch1 protein expression, accompanied by the downregulation of cyclin B1 and Bcl-2 in breast cancer cells	Suppress the proliferation of breast cancer, colon cancer, and neuroblastoma cells, while promoting apoptosis in colon cancer cells through the reversal of epithelial-mesenchymal transition (EMT)	Pan et al. (2012), Zhou et al. (2017)
Honokiol	Magnolia plants	Inhibit Notch pathway components in melanoma, liver cancer, and colon cancer cells, and effectively inhibit sphere formation and cell survival. Enhance the inhibitory effect on cancer cells *in vivo* when combined with ionizing radiation	Inhibit melanoma stem cells and demonstrate cytotoxic as well as cytostatic activity against malignant melanoma cells, and exert anti-tumor effects on liver and colon cancer	Kaushik et al. (2015), Kaushik et al. (2012), Ponnurangam et al. (2012)
Withaferin A	Ashwagandha	Suppress NICD1 expression in ovarian and colon cancer cell lines, and enhance the chemotherapy effect in ovarian cancer without adverse effects on normal colon cell lines	Suppress cell proliferation and trigger apoptosis in ovarian carcinoma cell lines CaOV3 and SKOV3, and target against colon carcinogenesis	Zhang et al. (2012), Koduru et al. (2010)
Hesperidin	Citrus fruits	Activate Notch1 signaling in ATC cells, thereby inducing the expression of apoptosis-related and differentiation markers	Induce apoptosis and cellular differentiation in anaplastic thyroid cancer (ATC) cells	Patel et al. (2014)
Phenethyl isothiocyanate	Cruciferous vegetables (trace amounts)	Effectively inhibit Notch levels in pancreatic cancer, HER2-positive breast cancer cells and ovarian cancer cells, thereby reducing cell proliferation and inducing apoptosis	Hamper the growth and development of HER2-positive breast and ovarian cancers through targeting their stem cell compartment, and inhibit proliferation and promote apoptosis in pancreatic cancer cells	Koscho et al. (2019), Stan et al. (2014)
Diallyl trisulfide	Garlic, onions and other Allium vegetables	Inhibit Notch1 and Hes1 expression in osteosarcoma cells, and inhibit Notch ligands and α-secretase activity in breast cancer cells	Inhibit proliferation, invasion and angiogenesis of osteosarcoma cells, contributing significantly to the regulation of tumor cell proliferation, invasion and angiogenesis in breast cancer	Kiesel and Stan (2017), Li et al. (2013)

## 3 Research results of Notch signaling pathway in different cancer types and limitations of current cancer treatments

### 3.1 Lung cancer

In non-small cell lung cancer (NSCLC), Notch1 plays a pivotal role in maintaining the stemness and proliferation of cancer stem cells (CSCs). Studies have shown that both Notch1 and its downstream effector protein Hes1 are significantly upregulated in CSCs of NSCLC compared with non-CSCs (Zhang et al., 2024). NO donors and NO production inhibitors can increase and inhibit Notch1 expression, respectively, and the regulatory effect of NO on Notch1 is not at the transcriptional level, but is achieved at the post-translational level, specifically by promoting the interaction between Notch1 and the deubiquitinating enzyme UCHL1 and reducing Notch1 ubiquitination. At the same time, NO-mediated S-nitrosylation of Notch1 can also inhibit its degradation, ultimately maintaining the stemness of CSCs (Zhang et al., 2024). Further studies have revealed that abnormal activation of Notch1 establishes a positive feedback cycle with the M2 polarization of tumor-associated macrophages (Sharif et al., 2020), while the role of Notch4 in tumor angiogenesis varies depending on vascular density (Costa et al., 2013).

Notch4 is particularly important in endothelial cells of lung cancer. In Calu-6 NSCLC tumors, Notch4 protein is predominantly localized in endothelial cells, and anti-Notch4 treatment leads to a significant reduction in tumor growth. However, in Lewis lung tumors, Notch4 is mainly expressed in non-vascular cell populations, and the corresponding anti-Notch4 treatment has no inhibitory effect on their growth, indicating that tumor endothelial Notch4 expression is critical for specific treatment-mediated tumor growth control (En et al., 2024). Anti-Notch4 treatment also significantly changes tumor endothelial cell gene expression, affecting genes related to angiogenesis and endothelial nitric oxide transport. Although no consistent changes in classical Notch signaling were observed, several pathways typically suppressed by Notch signaling, such as VEGF, thrombin, and Rho signaling, were upregulated following treatment. In addition, short-term Notch4 blockade can reduce tumor vascular perfusion, and long-term blockade can increase the number of tumor blood vessels and improve blood flow in the tumor (En et al., 2024).

### 3.2 Breast cancer

In breast cancer cells such as MCF-7, Notch pathway-related components including Notch1-4 receptors and their ligands Jagged1, Jagged2, and DLL1 are expressed, and the downstream target gene Hes1 is also actively expressed (Pandey et al., 2024). Resveratrol can dose-dependently reduce the mRNA and protein expression levels of Notch1 and Notch4 and Hes1, thereby inhibiting Notch pathway activity (Pandey et al., 2024). Inhibition of the Notch signaling pathway using γ-secretase inhibitors leads to increased activation of apoptosis-related proteins *via* cleavage of caspase-3, caspase-9 and PARP in MCF-7 cells. Conversely, overexpression of NICD1 can partially reverse resveratrol-induced apoptosis, indicating that the Notch pathway plays an anti-apoptotic role in breast cancer cells, and that resveratrol exerts a pro-apoptotic effect by inhibiting this pathway (Pandey et al., 2024).

DLL1 is highly expressed in estrogen receptor-positive (ER^+^) breast cancer and is linked to poor prognosis in patients. It participates in tumor growth and metastasis by promoting cell proliferation, maintaining breast cancer stem cells (BCSCs), and enhancing tumor angiogenesis (Sales-Dias et al., 2021). Specific anti-DLL1 IgG (such as IgG-69) screened by phage display technology can partially inhibit the DLL1-mediated Notch pathway activation, reduce the expression level of the Notch target gene Hey-L, suppress the proliferation of MCF-7 cells stimulated by rhDLL1-ECD-Fc, and reduce the BCSCs subpopulation in MCF-7 cells, with an effect comparable to that of the Notch inhibitor DAPT (Sales-Dias et al., 2021).

### 3.3 Gastrointestinal cancer

In gastric cancer, SGC-7901 cancer cells demonstrate markedly elevated mRNA and protein expression of LPA2 and Notch1 compared to normal gastric epithelial cells, with LPA shown to activate both LPA2 and Notch signaling pathways (Ren et al., 2019). Notch1 was identified as a mediator regulating EMT and cytoskeleton in SGC-7901 cells. LPA2 and Notch1 interact with each other, and LPA promotes this interaction (Ren et al., 2019). Analysis of gastric cancer patients found that the Notch1 and Notch2 mRNA levels in tumor tissues and peripheral blood mononuclear cells were significantly higher than those in healthy individuals, though no variations were observed across tumor-node-metastasis (TNM) stages (Yang et al., 2019). The percentages of CD4^+^CD25^+^CD127dim/−Tregs and Th17 cells in the peripheral blood of gastric cancer patients were markedly elevated compared to those in the healthy control group. Pharmacological inhibition using the γ-secretase inhibitor DAPT in gastric cancer patient-derived CD4^+^T cells suppresses Notch signaling-related molecules Hes1 and Hes5, modulates the transcription factors and secreted cytokines in Tregs and Th17 cells, and inhibits the inhibitory activity of Tregs (Yang et al., 2019). In the context of gastrointestinal cancer, some researchers have innovatively studied the regulatory effects of intestinal flora metabolites on Notch signaling and found that short-chain fatty acids affect the activity of the Notch pathway through epigenetic regulation (Dong et al., 2023).

In colorectal cancer, the expression of Golgi membrane protein 1 (GOLM1) is significantly reduced in tumor tissues relative to normal colon epithelium and is correlated with poor clinical outcomes. GOLM1-deficient mice are more sensitive to colitis and colon cancer (Pu et al., 2021). GOLM1 deficiency affects the differentiation of intestinal epithelial cells (IECs), increases the number of colon epithelial cells and decreases enteroendocrine cells. This deficiency inversely correlates with the downstream targets of the Notch signaling (Hes/Hey family genes), and increases the nuclear translocation of Notch2 (Pu et al., 2021). GOLM1 interacts with Notch2, and the cytoplasmic domain of GOLM1 binds to N2ICD, which is essential for maintaining the balance of Notch signaling in IECs (Pu et al., 2021). Treatment of GOLM1-ΔIEC mice with the Notch inhibitor DBZ can regulate intestinal cell differentiation and alleviate colitis symptoms, underscoring the necessity of balanced Notch signaling for intestinal homeostasis (Pu et al., 2021). In CSCs, the expression levels of SCD1 protein and mRNA are significantly higher than those in bulk cultured cells. SCD1 inhibition can induce apoptosis of CSCs and suppress the expression of Notch pathway-related genes. The mechanism is related to altering the levels of lipid metabolites to affect the composition of lipid rafts, thereby blocking the Notch signaling pathway (Yu et al., 2021).

### 3.4 Liver cancer

In hepatocellular carcinoma (HCC), EGFL8 expression is significantly reduced and is associated with multiple lesions, venous invasion, advanced TNM stage and poor prognosis of liver cancer (Wu et al., 2021). Functional studies revealed that EGFL8 overexpression suppressed HCC cell migration and invasion while modestly inducing apoptosis, though it exhibited no significant impact on proliferation *in vitro*. Consistent with these findings, *in vivo* experiments demonstrated a significantly reduced rate of pulmonary metastasis in EGFL8-overexpressing groups compared to controls (Wu et al., 2021). EGFL8 can regulate HCC cell migration, invasion and apoptosis through inhibition of the Notch signaling pathway, as evidenced by suppressed Notch-related protein expression in EGFL8-overexpressing cells and reciprocal upregulation of Notch-related proteins upon EGFL8 knockdown. Notably, pharmacological inhibition of Notch signaling using DAPT reverses the pro-metastatic and anti-apoptotic phenotypes of HCC cells caused by EGFL8 knockdown (Wu et al., 2021).

Unbiased screening and analysis of scRNA-seq and bulk RNA-seq data demonstrate a robust association between the Notch signaling pathway and SORT1, and pathway-related genes exhibit elevated expression and enrichment in the SORT1-high group (Ahn et al., 2024). After silencing SORT1 in Huh-7 and Hep3B cells, the key Notch pathway components and downstream effectors are significantly downregulated, establishing SORT1 as a regulator of Notch signaling activity. Further mechanistic studies reveal that SORT1 governs CD133 expression in HCC, positioning CD133 as a downstream effector of SORT1-mediated Notch pathway regulation (Ahn et al., 2024).

### 3.5 Hematologic tumors

In T-ALL, aberrant activation of the Notch signaling cascade is frequently observed. Approximately 50%–60% of patients harbor gain-of-function mutations in the Notch1 gene, and the Notch3 gene is frequently overexpressed. In some patients, the signaling pathway is overactivated due to inactivation mutations in the Notch negative regulator F-box and WD repeat domain-containing 7 (FBXW7) (Zhdanovskaya et al., 2022). Curcumin has an anti-proliferative effect on T-ALL cells by suppressing Notch pathway activity, downregulating associated proteins, promoting cell apoptosis and DNA damage, affecting the cell cycle, and regulating the transcriptional levels of some DNA damage repair-related genes (Zhdanovskaya et al., 2022). The curcumin derivative CD2066 has a stronger anti-proliferative activity against T-ALL cells at nanomolar concentrations, interferes with the activity of Notch1 and Notch3 receptors, promotes cell apoptosis, causes cell cycle arrest, and disrupts DNA repair mechanisms. Notch-driven leukemia cells are more sensitive to CD2066, and forced activation of Notch1 can partially protect cells from its anti-proliferative effects (Zhdanovskaya et al., 2022). Synergistic therapeutic efficacy is achieved when CD2066 is combined with the CDK1 inhibitor Ro3306, amplifying pro-apoptotic and anti-proliferative responses in KOPT-K1 cells (Zhdanovskaya et al., 2022).

In the *Drosophila* hematopoietic system, the Notch signaling pathway has different activities and functions in different regions. It is less active in the lymph gland cortical/intermediate zone cells, contributes to the formation and quantitative regulation of crystal cells during embryonic and larval hematopoiesis, and is also involved in the maintenance of progenitor cells and cell fate specification. Inhibition of Notch signals in related regions can induce the differentiation of lamellocytes to respond to infection (Luo et al., 2024). Activation of Notch signals can induce excessive differentiation of *Drosophila* progenitor cells, abnormal differentiation of lamellocytes, and non-apoptotic cell death, and also has context-dependent different effects on cell autophagy (Luo et al., 2024). In the *Drosophila* Ras^(v12)^ leukemia model, activation of Notch signals can alleviate cytokine storms, improve *Drosophila* survival, reduce circulating blood cell counts, and restore the expression of some cytokines and immune pathway target genes (Luo et al., 2024).

### 3.6 Limitations of cancer treatment

Although modern medicine has made significant progress in cancer treatment, cancer remains a leading cause of mortality, and treatment faces many challenges. Cancer exhibits profound molecular and cellular heterogeneity, which increases the difficulty of treatment (Guo et al., 2023). Cancer cells are prone to develop drug resistance through gene mutations, signaling pathway compensation, or microenvironmental adaptation, undermining the long-term efficacy of both targeted therapy and chemotherapy (Ortiz-Hernandez et al., 2024). Metastasis, the primary contributor to cancer-related mortality, remains inadequately addressed by current treatment modalities (Guan, 2015). Traditional therapies such as radiotherapy and chemotherapy cause severe toxic side effects due to their damage to normal tissues. Although immunotherapy has made certain breakthroughs, its effectiveness is constrained by patients’ immune status and tumor characteristics, while its high cost limits its widespread application (Wang F. et al., 2024). Therefore, utilizing naturally derived and inherently non-toxic food-derived functional factors with multiple bioactive properties for cancer prevention and therapy has emerged as a research focus.

## 4 Molecular mechanism of food functional ingredients regulating Notch signaling pathway

### 4.1 Polyphenol compounds

Epigallocatechin gallate (EGCG) extracted from tea leaves is the main bioactive substance of tea polyphenols and has broad application prospects in many fields such as tumor suppression and infection control (Park et al., 2021; Min and Kwon, 2014). Given the strong association between UC and colorectal cancer, the observed inhibition of Notch1, Hes1, and cleaved-Notch1 in lipopolysaccharide (LPS)-induced Caco-2 cells and DSS-induced UC mice provides indirect evidence supporting EGCG’s potential involvement in intestinal tumorigenesis *via* Notch signaling (Li, 2022). EGCG also modulates other pathways such as NF-κB and MAPKs, which intersect with Notch signaling, suggesting broader participation in cancer-related signaling networks (Min and Kwon, 2014). In nasopharyngeal carcinoma CNE2 cells, EGCG upregulates miR-34a, which directly targets Notch1, thereby suppressing cancer cell proliferation, migration, and invasion (Li B. B. et al., 2017). Overall, EGCG can directly or indirectly inhibit aberrant Notch activation and downregulate core components such as Notch1 and Hes1, ultimately suppressing cancer progression. Evidence from intestinal-related cancer models and nasopharyngeal carcinoma supports this mechanism.

Resveratrol, a non-flavonoid polyphenol derived from the rhizome of *Polygonum cuspidatum*, is an effective anti-cancer bioactive substance that can trigger cancer cell apoptosis (Pandey et al., 2024). Notably, *Polygonum cuspidatum* is also a commonly used traditional Chinese medicinal material (Huzhang), and thus resveratrol represents both a food-derived functional component and an important active constituent of traditional herbal medicine (Wang et al., 2002). It suppresses Notch signaling by downregulating core components (Notch1, Notch4, Jagged1), reducing anti-apoptotic Bcl-2 expression, and elevating pro-apoptotic Bax levels. It can also block the Notch1-Hes1 axis, reduce the transcriptional activity of downstream target genes, induce mitochondrial pathway apoptosis *via* increasing the Bax/Bcl-2 ratios, inducing mitochondrial membrane depolarization, promoting cytochrome c release, and triggering caspase cascade activation (Kaveh Zenjanab et al., 2024). Studies in HCT116 colorectal cancer cells demonstrate its capacity to inhibit proliferation, migration, and survival through Notch pathway suppression (Ren et al., 2023). Interestingly, resveratrol also exhibits a unique biphasic regulatory property: at low concentrations it enhances NICD degradation *via* the SIRT1-dependent pathway, whereas at high concentrations it can directly bind to the γ-secretase active site (Guarani et al., 2011).

Curcumin, a natural polyphenol extracted from turmeric rhizomes, can directly bind to Notch1 receptors to prevent them from being activated, or indirectly affect Notch pathway conduction by inhibiting the activity of related protein kinases such as Akt and ERK (Gupta et al., 2013). Meanwhile, turmeric (Jianghuang) has long been used as a traditional Chinese medicinal material, so curcumin likewise serves as both a food functional component and a key bioactive compound in traditional herbal medicine (Aggarwal and Sung, 2009). In models of pancreatic cancer and liver cancer, it can downregulate the Notch pathway, inhibit tumor progression/metastasis, enhance chemotherapeutic efficacy and reduce drug resistance in tumor cells (Tang and Cao, 2022; Mimeault and Batra, 2011; Peng et al., 2019). Curcumin demonstrates cytotoxicity in SW480 colon cancer cells *via* downregulation of Notch1/Hes1 expression (Liu et al., 2015). Curcumin derivatives exert multimodal Notch pathway inhibition by suppressing Notch1 receptor expression, blocking anti-apoptotic downstream effects, activating the mitochondrial apoptosis pathway, enhancing DNA damage, and inhibiting repair functions (Zhdanovskaya et al., 2022). Curcumin-mediated photodynamic therapy (PDT) significantly downregulates Notch1 and its downstream effectors NF-κB and VEGF in both *in vitro* and *in vivo* cervical cancer models (He et al., 2019). Moreover, when combined with the γ-secretase inhibitor DAPT, curcumin-PDT exhibits synergistic anti-proliferative and pro-apoptotic effects, further confirming the Notch signaling pathway as a key therapeutic target of curcumin-based anticancer intervention (He et al., 2019).

Quercetin, widely found in fruits, vegetables, and tea, can inhibit Notch pathway activity across multiple malignancies (breast, lung, and colon). It reduces NICD production by inhibiting γ-secretase activity, downregulates Hes1/Hey1, and induces cancer cell apoptosis while suppressing proliferation/migration (Li et al., 2020). For example, in breast cancer models, quercetin decreases Notch1 protein levels, arrests cell cycle progression, and increases apoptosis (Cao et al., 2018; Jiang et al., 2021). Quercetin-3-methyl ether can inhibit Notch1 expression in MCF-7 and MDA-MB-231 cells and downregulate protein markers related to cell cycle and apoptosis (Cao et al., 2018). Pectin/chitosan-encapsulated quercetin microspheres can relieve the symptoms of inflammatory bowel disease (IBD) by inhibiting the Notch pathway, reshaping the inflammatory microenvironment, and repairing the intestinal mucosal barrier (Gan et al., 2024). In colon CSCs, quercetin combined with radiotherapy can reduce the levels of Jagged1 protein, γ-secretase complex components, and cleaved Notch1, upregulate miR-200b-3p, inhibit Notch signaling, change the division pattern of CSCs, and impair tumor regeneration (Kaveh Zenjanab et al., 2024). In addition, quercetin can regulate Th cell responses, indirectly affect the Notch signaling pathway by inhibiting the NLRP3 inflammasome, and enhance colorectal cancer radiosensitivity *via* Notch1 pathway blockade. These combined actions culminate in the downregulation of cancer stem cell marker expression (Das et al., 2023).

Rutin is a flavonoid widely distributed in plants. It can reduce Notch1 gene transcription and synthesis, downregulate Hes1 expression, and by regulating Notch1 and Hes1, not only inhibit tumor cell proliferation but also activate apoptosis pathways. Its anticancer efficacy is further amplified *via* modulating cross-talk between Notch signaling and other signaling cascades, including the PI3K/Akt and MAPK pathways, which indirectly attenuate Notch signaling. *In vitro* experiments using Caski cervical cancer cells have shown that rutin inhibits proliferation and induces apoptosis in a dose-dependent manner, accompanied by downregulated Notch1 and Hes1 expression (Khan et al., 2021).

### 4.2 Terpenoids

Ursolic acid, abundant in the peels of fruits such as apples and hawthorns, can suppress Notch pathway activation by downregulating the expression of receptors and ligands and reducing the nuclear translocation of NICD, thereby curbing cancer cell proliferation and invasion *via* the Notch pathway. In prostate cancer and gastric cancer cells, it can inhibit the Notch signaling pathway, suppress cell growth, and upregulate proteins associated with apoptosis (Mallepogu et al., 2023). In liver fibrosis research, ursolic acid can inhibit the expression of Notch3 signaling-related molecules in TGF-β1-activated hepatic stellate cells (HSCs). Consistently, silencing Notch3 can regulate the biological behavior of HSCs and the levels of fibrosis-related indicators. *In vivo* experiments have also confirmed that ursolic acid can improve the liver environment in fibrotic mice and inhibit liver fibrosis progression by regulating the Notch3/NOX4 signaling pathway (Zhang, 2023). In addition to modulating the Notch pathway, its anticancer activities involve inducing apoptosis, causing cell cycle arrest, and inhibiting both metastasis and angiogenesis (Chan et al., 2019).

Triptolide, a diterpenoid compound from *Tripterygium wilfordii*, can disrupt the crosstalk between the Notch signaling pathway and other pathways such as Wnt, thereby modulating the stemness and differentiation of cancer cells. In leukemia cells, triptolide suppresses the Notch pathway, reduces the proportion of leukemia stem cells, and induces cell differentiation (Wu et al., 2023; Xu et al., 2023). Studies have shown that the monomer drug triptolide plays a crucial role in the regulation of the Notch pathway that is suppressed by methotrexate (MTX) (Wu et al., 2023). Specifically, triptolide can mitigate MTX-induced suppression of the Notch pathway, which is manifested in upregulating Notch1 and other Notch-related proteins, downregulating inhibitory factors (e.g., Numb), and altering the mRNA levels of Notch pathway members (Wu et al., 2023). Triptolide can upregulate Notch1 protein levels while downregulating Numb protein expression, and simultaneously increase the mRNA levels of other Notch pathway members. As a result, triptolide partially restores Notch1 protein levels suppressed by MTX and reverses the MTX-induced downregulation of other Notch-related genes (Wu et al., 2023). This regulatory effect ultimately limits the inhibitory impact of MTX on bone marrow cell proliferation, primarily through downregulation of Numb and subsequent activation of the Notch signaling pathway (Wu et al., 2023).

### 4.3 Sulfur-containing compounds

Allicin extracted from garlic can affect the activity of γ-secretase by regulating the redox state, thereby influencing the Notch signaling pathway. In gastric and colon cancer cells, it suppresses Notch pathway activation, promotes apoptosis of cancer cells, inhibits tumor growth, and prevents cancer occurrence (Huang et al., 2016; Kumari et al., 2024; Guilmeau, 2012). In addition, by inhibiting α-secretase (a protease involved in Notch processing), allicin can suppress the abnormal activation of the Notch signaling pathway and prevent breast cancer (Kiesel and Stan, 2017). Diallyl trisulfide (DATS), a major metabolic derivative of garlic, similarly targets the Notch pathway but primarily through epigenetic and post-transcriptional mechanisms. DATS suppresses Notch signaling by upregulating tumor-suppressive microRNAs (miR-34a, miR-143, miR-145, and miR-200b/c) and downregulating Notch1 and its downstream target genes (Li et al., 2013; Kim et al., 2016). Through this mechanism, DATS inhibits cancer cell proliferation, invasion, angiogenesis, and stemness while inducing cell cycle arrest and apoptosis in osteosarcoma and breast cancer models (Li et al., 2013; Pandey et al., 2023). Moreover, DATS can modulate a reciprocal feedback loop between Notch1 and microRNAs, in which Notch1 inhibition further enhances the expression of tumor-suppressive microRNAs, thereby amplifying its antitumor effects (Li et al., 2013; Kim et al., 2016).

Sulforaphane in cruciferous vegetables such as broccoli can reduce cancer cell proliferation, migration and invasion, and enhance cell sensitivity to radiotherapy and chemotherapy. In SW480 cells, sulforaphane treatment (1, 2, 5 μmol/L) significantly reduced the expression of proteins related to the Notch signaling pathway (Notch1, Notch3, Hes1), as well as markers of cell proliferation (Ki-67, PCNA) and migration/invasion (MMP-9), in a dose-dependent manner, and increased E-cadherin expression. This is because sulforaphane inhibits the Notch signaling pathway, blocks the nuclear translocation of NICD, affects downstream gene expression, and thereby indirectly regulates these protein levels and ultimately suppress cell proliferation, invasion and migration (Xing et al., 2021). Additionally, sulforaphane has a regulatory effect on the ZNF217/Notch1 axis, which can reduce the levels of ZNF217 and Notch pathway-related proteins in a dose-dependent manner. Both *in vitro* and *in vivo* studies have shown that overexpression of ZNF217 can restore the self-renewal ability inhibited by sulforaphane by reactivating the Notch pathway. Sulforaphane also inhibits Notch signaling and the expression of colorectal cancer stem cell markers, and ZNF217 overexpression can reverse this inhibition, indicating that sulforaphane targets the ZNF217/Notch1 axis to modulate Notch signaling and inhibit the stem-like properties of colorectal cancer cells (Wang M. et al., 2024).

### 4.4 Vitamins

As an active metabolite of vitamin A, retinoic acid plays a vital role in regulating cell differentiation and proliferation. It can indirectly influence the expression of genes associated with the Notch pathway by binding to nuclear receptors, thereby inducing cancer cell differentiation. Part of the mechanism is to inhibit Notch pathway activity, reduce the expression of target genes, and induce cancer cell differentiation toward normal phenotypes (Chin et al., 2022). In SACC cells, Notch1 knockdown can cause changes in the expression of RA signal-related molecules and downregulate MYB and MYC through the RA pathway. *In vitro* studies show that combining all-trans retinoic acid (ATRA) with the γ-secretase inhibitor DAPT can inhibit cell proliferation and invasion more effectively than using either agent alone. *In vivo* experiments show that low-dose ATRA can more significantly inhibit lung metastasis. Retinoic acid can regulate the Notch signaling pathway by modulating Notch1 and its related molecules, thereby affecting downstream genes and processes such as EMT, and ultimately reducing lung metastasis (Zhou et al., 2023).

Cellular experiments have confirmed that vitamin D can inhibit the proliferation, migration, and invasion of colorectal cancer cells (SW480) and promote their apoptosis by downregulating the Notch1 pathway (Liu et al., 2023). In SW480 cells treated with vitamin D, the protein expression levels of Notch1, Cleaved-Notch1, and its downstream target gene Hes1 were significantly reduced, while overexpression of Notch1 reversed the anti-cancer effects of vitamin D, fully demonstrating that Notch signaling activity was inhibited (Liu et al., 2023). Furthermore, in human colon normal organoids, vitamin D can counteract Notch inhibitor (DBZ)-induced goblet cell differentiation, indirectly regulating Notch pathway-mediated cell fate determination by maintaining stem cell characteristics and weakening the expression of differentiation-related genes (Bustamante-Madrid et al., 2024). Vitamin D may exert its anti-colorectal cancer effects by regulating the cleavage and processing of Notch receptors or the transcription of downstream target genes, thereby modulating the Notch signaling pathway (Liu et al., 2023; Bustamante-Madrid et al., 2024).

### 4.5 Minerals

As a trace element, Selenium (Se) plays a crucial role in maintaining human health and has antioxidant and anti-cancer effects. Selenium compounds can modulate the activity and stability of Notch pathway-related proteins by regulating the intracellular redox state. In breast and liver cancer cells, these compounds can suppress the Notch pathway to diminish proliferation, increase apoptosis, and enhance immune function, indirectly inhibiting tumor growth and metastasis. The immunomodulatory effects of Se are linked to its regulation of Notch signaling in immune cells (Powers et al., 2021). Se can also regulate the Notch pathway by changing the intracellular environment, thereby affecting key cellular processes such as differentiation, cell fate determination, apoptosis, and cell growth (Knight et al., 2016).

Zinc ions are crucial regulators of cellular signal transduction, including the Notch pathway. Appropriate zinc levels are required to maintain the structural integrity and functional activation of Notch receptors, whereas zinc deficiency or excess can disrupt Notch signal and alter the biological behavior of cancer cells (Guo et al., 2024b; Bafaro et al., 2017; Costa et al., 2023). In addition to direct effects on signaling components, zinc also regulates Notch activity through zinc finger proteins, whose structure and transcriptional functions depend on zinc coordination. Zinc finger protein 521 (ZNF521) positively regulates Notch signaling by forming a functional complex with p300, thereby enhancing the expression of Notch-related components such as nuclear factor erythroid 2—related factor 2 (NRF2); silencing ZNF521 impairs spheroid formation and stem cell frequency in ES-2 cells (Scicchitano et al., 2023). In contrast, zinc finger protein 671 (ZNF671) is negatively correlated with Notch signaling in colorectal cancer. Overexpression of ZNF671 suppresses the expression of Notch1 and its downstream targets, whereas ZNF671 silencing enhances Notch pathway activation, promoting cancer cell proliferation and invasion—effects that can be reversed by Notch inhibition (Wang et al., 2023). Similarly, zinc finger RNA-binding protein (ZFR) is related to the Notch1 signaling pathway in NSCLC cells. Downregulation of ZFR can reduce the expression of Notch1 and affect the migration and invasion ability of NSCLC cells, while ZFR overexpression exerts opposite effects that are dependent on Notch1 activity (Zhang et al., 2017).

### 4.6 Other ingredients

Lycopene is a natural carotenoid with well-documented antioxidant properties. Accumulating evidence suggests that lycopene can modulate cancer-related signaling pathways, including the Notch pathway. In breast cancer and colorectal cancer cells, lycopene significantly downregulates the expression of Notch1 and its ligand Jagged1, blocks nuclear translocation of the NICD, and subsequently suppresses Hes1 expression, leading to inhibition of cell proliferation and induction of apoptosis (Yang et al., 2024). Furthermore, in nude mouse xenograft models, lycopene reduces tumor volume and exhibits synergistic antitumor effects when combined with γ-secretase inhibitors, providing *in vivo* evidence that lycopene exerts anticancer activity through modulation of the Notch signaling pathway (Yang et al., 2024).

Short-chain fatty acids, such as butyrate, can affect the expression of genes related to the Notch signaling pathway by modifying histone acetylation. In the LPS-treated group, the expression of Notch pathway-related genes changed over time; however, the LPS and butyrate co-treatment group could inhibit this time-dependent bidirectional fluctuation in gene expression (i.e., downregulation at 6 h and upregulation at 12 h). This indicated that butyrate affected the expression of genes related to the Notch signaling pathway by regulating histone acetylation modification, and participated in the immune regulation process of *Pinctada martensii* by modulating the Notch pathway. Specifically, its modulation of the Notch pathway acts synergistically with other immune-related pathways, including NF-κB and TNF signaling, affecting the inflammatory response of macrophages. It can also change the cell proliferation state by modulating Notch activity and inhibiting LPS-induced hematopoietic cell proliferation (Yang et al., 2023).

Analysis using bioinformatics tools revealed that the target proteins and their associated signaling pathways of the upregulated microRNAs in the EPA-treated group were concentrated in the MAPK and Notch signaling pathways. These microRNAs could affect Notch pathway activity by targeting proteins involved in the Notch signaling cascade. The downstream proteins of the differentially expressed microRNAs were associated with functions such as cell metabolism and the cell cycle. Furthermore, the signaling pathways targeted by these microRNAs were associated with the maturation and function of dendritic cells. The microRNAs regulated by ω-3 polyunsaturated fatty acids could participate in dendritic cell maturation and the regulation of related cell functions by affecting the Notch signaling pathway (Gao, 2017).

**FIGURE 2:**
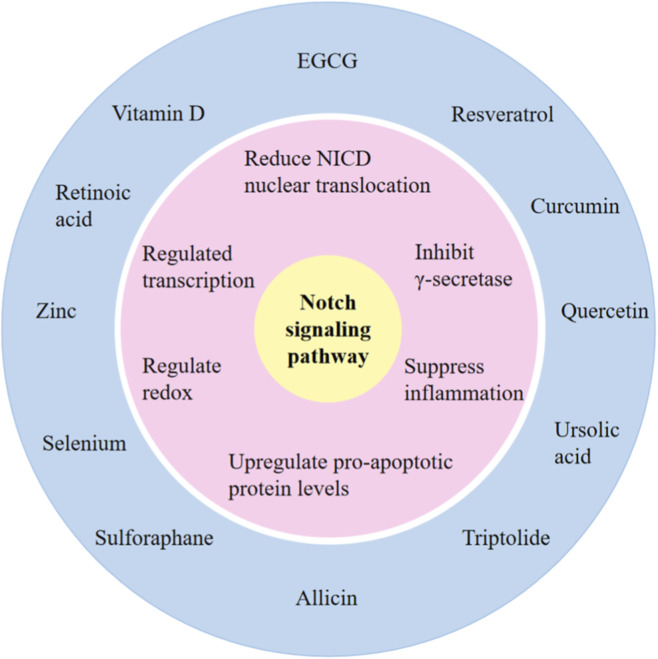
Molecular mechanism of food functional ingredients regulating Notch signaling pathway. This figure summarizes representative food functional ingredients (outer circle) and their major regulatory actions on the Notch signaling pathway (middle circle), including inhibition of γ-secretase, reduction of NICD nuclear translocation, modulation of transcription, redox regulation, suppression of inflammation, and upregulation of pro-apoptotic proteins.

In summary, functional components of different food categories can target various molecular nodes of the Notch signaling pathway, forming a multidimensional, multi-target regulatory network to intervene in tumor development and progression (Figure 2). Furthermore, the regulation of the Notch pathway by these components does not occur in isolation, but rather interacts with other anti-cancer signaling pathways, constructing a synergistic molecular network for cancer suppression. Elucidating these mechanisms not only provides molecular-level theoretical support for understanding the anti-cancer effects of functional food components but also lays a crucial foundation for the subsequent development of Notch pathway-targeted strategies based on dietary intervention and the design of personalized anti-cancer regimens involving multiple components. It should be noted that some food-derived compounds possess strong pharmacological activity and may interfere with the normal tissue regeneration process dependent on Notch signaling. Therefore, the safety of their long-term use requires further verification.

## 5 Animal experiments-evidence on the role of food functional factors in treating cancer by intervening in the Notch pathway

The researchers used BALB/c nude mice to construct a pancreatic cancer xenograft model and inoculated Capan-1/DDP cells into the flanks of nude mice to establish pancreatic cancer xenografts (Zhang et al., 2023). The experiment was divided into four groups: a model group, an IL-6 group, a cisplatin + IL-6 group, and an IL-6+Lentinan (LNT) group. The drugs were intraperitoneally administered to mice in each experimental group once every 3 days at the doses specified in the original study (Zhao et al., 2018), and the tumor size was measured weekly (Zhang et al., 2023). The results showed that the tumor volume and weight in the IL-6 group increased significantly, confirming that IL-6 promoted tumor proliferation. In contrast, the IL-6+LNT group showed a marked reduction in both tumor volume and weight, indicating that LNT could effectively inhibit the tumorigenic effect of IL-6, thereby suppressing tumorigenesis (Zhang et al., 2023). In tumor tissues, the expression levels of Notch1 and Hes1 were dramatically upregulated in the IL-6 group. However, following treatment with LNT (in the IL-6+LNT group) and cisplatin (in the cisplatin + IL-6 group), the expression levels of p-STAT3/STAT3, Notch1, and Hes1 in both groups were significantly downregulated, indicating that LNT and cisplatin can mitigate the tumorigenic effects of IL-6, effectively suppress the IL-6/STAT3/Notch signaling pathway, and thus inhibit cancer cell proliferation (Zhang et al., 2023).

Chlorogenic acid is derived from various food sources such as coffee, honeysuckle, eucommia, apples, and pears. Meanwhile, some researchers used 4-week-old healthy SPF-grade male BALB/c-nu nude mice and implanted A549 lung cancer cells subcutaneously into their flanks to establish a lung cancer-bearing model (Li W. et al., 2017). The experimental group was injected with chlorogenic acid solution in the tumor area every day, while the control group was administered an equal volume of saline for 4 weeks (Li W. et al., 2017). The results showed that both tumor size and weight were significantly reduced in the experimental group compared to the control group. Moreover, no metastases were observed in the experimental group, while there were micrometastases in the lungs of the control group, which means that chlorogenic acid markedly suppresses tumor growth and proliferation (Li W. et al., 2017). Real-time quantitative PCR analysis revealed significantly lower mRNA expression levels of Notch1 ligand DLL4, VEGF, and downstream effector molecules of the Notch1 signaling pathway, namely, Hes1 and Hey1, in the experimental group compared to the control group, suggesting that chlorogenic acid can affect the Notch1 signaling pathway by downregulating the expression of these molecules. Western blotting analysis further confirmed that Notch1 protein levels were significantly decreased in the experimental group, while the expression of p-PTEN and total PTEN proteins was increased, and p-Akt levels were reduced, indicating that the Notch1 pathway modulates the tumor suppressor gene PTEN through cross-regulation with the PI3K/AKT pathway, thereby affecting the biological function of lung cancer cells (Li W. et al., 2017).

These *in vivo* animal experiments further verified the significant effect of food functional ingredients on regulating the Notch pathway and inhibiting tumor growth and metastasis, and strongly facilitated their translation from theoretical research to clinical application. The findings of *in vitro* cell experiments were reconfirmed at the animal level (e.g., tumor growth and metastasis), providing strong evidence for the clinical potential of food functional ingredients in cancer therapy, and thus greatly enhancing their feasibility and potential value in clinical practice.

## 6 Conclusion and prospects

This review deeply explores the complex role of the Notch signaling pathway in cancer, as well as the regulatory mechanisms and potential anti-cancer effects of food functional ingredients on this pathway. The Notch signaling pathway is critically involved in maintaining normal cellular physiological processes, as well as the occurrence and development of cancer. Its abnormal activation or inhibition is closely associated with tumor cell proliferation, apoptosis, invasion, and metastasis, as well as regulating the tumor microenvironment. As natural bioactive substances, food functional ingredients include polyphenols, terpenoids, sulfur-containing compounds, vitamins, minerals, carotenoids, dietary fiber, and their metabolites. They precisely regulate the Notch signaling pathway through different molecular mechanisms and exhibit significant anti-cancer activity.

To enhance the hierarchy of evidence, current findings can be categorized into *in vitro*, animal, and human studies. *In vitro* studies provide mechanistic insights into how these ingredients modulate Notch-related proteins, gene expression, and cancer cell behaviors. Animal studies further validate these mechanisms by demonstrating inhibition of tumor growth and metastasis *in vivo*. However, clinical evidence remains scarce, and existing human studies offer only preliminary indications of relevance, highlighting the need for more robust clinical validation.

Dose relevance and bioavailability challenges remain major barriers to clinical translation. Many effective concentrations observed in cell culture exceed levels achievable through normal dietary intake, and even animal doses do not always correspond to physiologically attainable human levels. Moreover, numerous compounds—especially polyphenols, terpenoids, and carotenoids—exhibit poor absorption, rapid metabolism, and low systemic stability. Improving bioavailability through optimized formulations, delivery systems, or synergistic combinations is therefore critical for bridging mechanistic findings with clinical applicability.

Looking ahead, several priority directions should be emphasized. Among the compounds reviewed, polyphenols such as EGCG and resveratrol, ω-3 polyunsaturated fatty acids, and selected terpenoids show the most consistent mechanistic and preclinical evidence and should be prioritized for further investigation. Future research should integrate pharmacokinetic analyses, dose-response evaluations, and controlled animal studies, followed by early-phase clinical trials to determine feasible dosing ranges and assess whether biologically active concentrations can be achieved in humans.

Despite the promising progress summarized above, three key challenges must be explicitly addressed to advance the translational potential of this field.

First, the context-dependent complexity of Notch pathway modulation remains poorly understood. As highlighted in earlier sections, Notch exhibits dual roles (pro-tumor in T-ALL (Zhang et al., 2025) vs. tumor-suppressive in colorectal cancer (Zhdanovskaya et al., 2022; He et al., 2016)), yet most studies on food functional ingredients focus on general Notch inhibition without distinguishing among cancer subtypes. This lack of subtype-specific mechanistic insights may lead to inconsistent efficacy-for example, resveratrol inhibits Notch1 (a key pro-tumor factor in breast cancer) to suppress breast cancer growth (Pandey et al., 2024; Dong et al., 2020; Lu and Serrero, 1999), underscoring the need for subtype-specific research.

Second, standardization of efficacy evaluation criteria is lacking. Current *in vitro* and *in vivo* studies use divergent biomarkers to assess Notch pathway activity: some rely on Hes1/Hey1 mRNA expression, others on NICD protein levels, and still others on downstream functional phenotypes. This inconsistency makes cross-study comparisons difficult-for instance, two separate studies on sulforaphane’s Notch-inhibiting effect—one in SW480 cells (Liu et al., 2020) and one in HCT116 cells (Wang M. et al., 2024)—both confirmed Notch inhibition but used different biomarkers, complicating cross-study interpretation.

Third, long-term safety of food functional ingredients in Notch modulation needs further verification. Preclinical studies primarily evaluate short-term (4–8 weeks) anti-tumor effects, but long-term administration may disrupt Notch-dependent normal physiological processes-such as ursolic acid’s potential to inhibit Notch3 in hepatic stellate cells (Zhang, 2023), which could interfere with liver tissue repair if used chronically. Additionally, the interaction between food functional ingredients and traditional cancer therapies (e.g., whether quercetin affects chemotherapy-induced Notch inhibition (Das et al., 2023)) remains largely unexplored, posing potential risks for clinical combination use.

In short, the study of Notch signaling pathway and food functional ingredients has opened up a promising new path for cancer treatment. Addressing these challenges is crucial for unlocking the full anticancer potential of food functional ingredients through the regulation of the Notch signaling pathway, and this field deserves the attention and in-depth exploration of scientific researchers, clinicians and the general public.
